# The Book World of Medicine and Science

**Published:** 1896-01-18

**Authors:** 


### The Book World of Medicine and Science.

Toxin. By Ouida. (London: T. Fisher Unwin, 1895. Price 1s. 6d.)

But for its pretty cover and the name of its gifted, if somewhat eccentric, authoress, this book does not appear likely to interest many people. As in temperance novels, where all the people who drink tea are good and those who taste wine come to a bad end, the motive is too transparent. Ordinary readers are a little tired of anti-vivisectors, and ignorant descriptions of disembowelled dogs are not specially attractive. The book is really a somewhat malignant attempt to throw obloquy on physiologists and all their doings. Ignorance and science are contrasted, much to the advantage of the former. We are only introduced to three characters, of whom two are ignorant, but lovable and fair, while the third, the English surgeon and physiologist, is scientific and at the same time hateful and of fiendish wickedness. Quite like a little temperance tract! This wicked man is a common poisoner actuated by common motives, and the only peculiarity about him is that he brews toxin instead of ordinary poison, and when his victim is suffering from diphtheria, injects him with this instead of with anti-toxin, and so ensures his death. Of course, all this is common villany enough, and the whole interest lies in the suggestion that of such mould are English physiologists and those who use anti-toxin. As a word-painter Ouida's power is well-known, and her picture of a death from diphtheria is sufficiently striking to be worth reading by those who protest against the use of animals for mitigating the sufferings of man, however much they be used for mitigating hunger and for other purposes. Here it is: "His head was thrown back on the pillows; his eyes were staring but sightless; his face was pallid and looked blue round the mouth and about the temples. He was now straining for breath, like a horse fallen on the road, blown and broken. . . The pure light of earliest daybreak was in the whole of the vast chamber. It shone on that ghastly sight, a man dying in his youth, struggling and straining for a breath of air, fighting against suffocation. . . . The poisoned growth filled every chink of the air passages, as though they were tubes mortared up and closed hermetically. His face grew purple and tumid, his eyes started from their sockets, his arms waved wildly, beckoning in space; he had no sense left except the mere instinctive mechanical effort to gasp for the air he was never to breathe again. The five persons around him stood in silence while the stifled sobs of the nun were heard. . . . The minutes went on; the nuns sank on their knees; the one who had wept hid her face on the coverlet of the bed. All which had been so lately the youth, the form, the vitality of Adrianis, wrestled with death as a young lion tears at the walls of the den which imprisons him. The terrible choking sounds roared through the air to which his closed throat could not open. Blood foamed in froth from his lips, which were curled over the white teeth and were cracked and blue. His eyes starting from their orbits had no sight. . . . Suddenly the convulsions ceased. 'He is out of pain,' said one of the Venetians in a solemn and hushed voice. 'He is dead,' said Damer." This is Ouida's description of a death from diphtheria, and we wish to heaven we could say it was not a true one; for we do not forget that from diphtheria 2,637 persons died in London alone during the year 1894, and the disease is still increasing. The thought is horrible, and yet people are found who, while protesting against what they are pleased to call vivisection—the injection of a few rabbits which otherwise would be shot or worried, or the bleeding of a few horses, kept in comfort all the time, which otherwise would long since have gone to the knacker's yard—are apparently willing that their fellow creatures should die such deaths. Surely it is a sign of either crass ignorance or wicked indifference to human life that people should be found to oppose the efforts of honest men of science to cure such a disease as this.

The London Homœopathic Hospital Reports. Edited by G. Burford, M.B., C. Knox Shaw, and Byres Moir, M.D. Vol. IV., 1894. (London: E. Gould and Son. Pp. 187. Ten illustrations and plates.)

It is a pleasure to see from the report that much good work is being carried on at the London Homœopathic Hospital, for though we cannot pretend to favour the views and practice that is peculiarly associated with the school of thought which this hospital represents, the report before us is highly creditable to those from whom we differ. Dr. Bayes contributes an able and scientific analysis of the symptoms produced by anacardium. Mr. W. Epps reports two cases of Bazin's disease, of which he gives excellent coloured plates. The clinical phenomena of appendicitis are well described by Mr. Knox Shaw, while Dr. Byers Moir writes an excellent article on tracheotomy in diphtheria, and we congratulate him on obtaining 70 per cent. of recoveries in ten cases operated on, none over six years of age. Mr. Dudley Wright is interesting on diseases of the jaws, with two plates. Three plates are given in illustration of Dr. Neatby's case of epithelioma. Many other articles deserve notice. Altogether, the editors are fortunate in being so well supported in their efforts to produce an interesting volume.

